# Transadmittance Type Universal Current-Mode Biquad Filter Using VDTAs

**DOI:** 10.1155/2014/762845

**Published:** 2014-08-19

**Authors:** Dinesh Prasad, Mayank Srivastava, D. R. Bhaskar

**Affiliations:** ^1^Department of Electronics and Communication Engineering, Faculty of Engineering and Technology, Jamia Millia Islamia, New Delhi 110025, India; ^2^Department of Electronics and Communication Engineering, Amity School of Engineering and Technology, Amity University, Sector 125, Noida 201313, Uttar Pradesh, India

## Abstract

A new resistorless single-input-multi-output (SIMO) universal transadmittance (TA) type filter employing two voltage differencing transconductance amplifiers (VDTA) and two grounded capacitors is proposed. The proposed topology realizes simultaneously low pass (LP), high pass (HP), and band pass (BP) filter functions. Band rejects (BR) and all pass (AP) filters are also realizable through appropriate connections of currents. The proposed configuration also offers independent control of natural angular frequency (*ω*_0_) and bandwidth (BW) and low active and passive sensitivities. The workability of proposed configuration has been demonstrated through PSPICE simulations with TSMC CMOS 0.18 *μ*m process parameters.

## 1. Introduction

Multifunction filters are getting significant attention in current analog IC design due to their versatility as the same configuration can be used for various filter responses. In the literature, SIMO type active filters employing different active building blocks/devices are available in both current mode (CM) and voltage mode (VM). The advantages and utility of TA type filters have been highlighted in [1]. There are few research publications which have been published dealing with the realisation of TA type filters [1–6].

VDTA is one of the active elements out of those introduced in [7]. Owing to its flexibility and versatility, compared to other active building blocks/devices, several VDTA-based applications have been reported in the literature [8–13]. In [8], Yeşil et al. proposed a simple CMOS realization of VDTA and its application as RF filter and double tuned amplifier. A SIMO VM biquad filter has been reported in [9]. In [11], an electronically controllable explicit current-mode sinusoidal oscillator using single VDTA and in [12] an electronically controllable fully uncoupled sinusoidal oscillator using two VDTAs have been presented. A universal current-mode biquad filter using single VDTA has also been reported in [13] and grounded and floating inductance circuits using single/two VDTAs and one grounded capacitor have been proposed in [10].

In this communication, we present a new configuration for realizing TA universal biquadratic filter with one input and three outputs. The proposed new circuit offers various features such as employment of only two VDTAs, two grounded capacitors, simultaneous realization of LP, HP, and BP filtering responses without changing circuit topology, independent control of *ω*_0_ and BW, and low active and passive sensitivities. BR and AP filter responses are also obtainable through proper connections of currents. The workability of the proposed TA filter has been verified by SPICE simulations with TSMC CMOS 0.18 *μ*m process parameters.

## 2. The Proposed Transadmittance Filter

**Figure 1:**
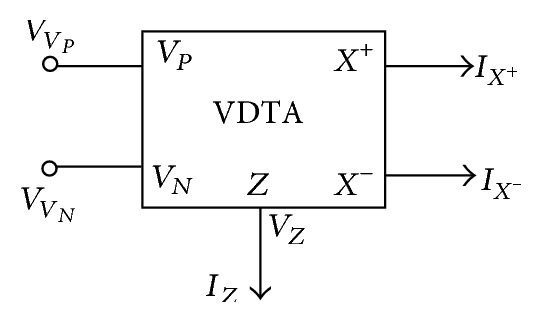
The symbolic notation of VDTA.

The symbolic notation of the VDTA is shown in Figure 1, where *V*_*P*_ and *V*_*N*_ are input terminals and *Z*, *X* ^+^, and *X* ^−^ are output terminals, having high impedance values [8]. The terminal equations can be described by the following set of equations:

(1) $$\begin{array}{c} \left[\begin{array}{c} I_{Z}\\ I_{X^{+}}\\ I_{X^{-}} \end{array}\right]=\left[\begin{array}{ccc} g_{m_{1}} & -g_{m_{1}} & 0\\ 0 & 0 & g_{m_{2}}\\ 0 & 0 & -g_{m_{2}} \end{array}\right]\left[\begin{array}{c} V_{V_{P}}\\ V_{V_{N}}\\ V_{Z} \end{array}\right]. \end{array}$$

**Figure 2:**
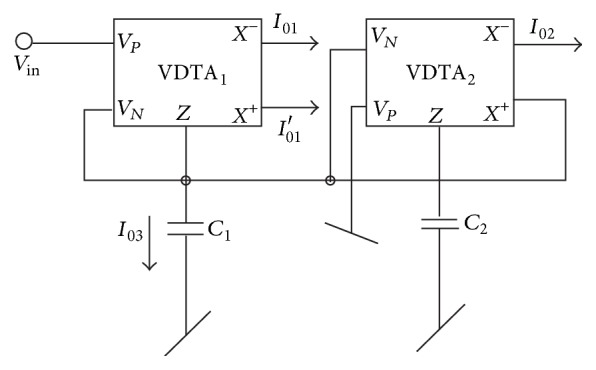
The proposed TA biquad filter.

The proposed TA filter configuration is shown in Figure 2.

Assuming ideal VDTAs, a routine analysis of the circuit shown in Figure 2 yields the following current transfer functions:

(2) T1(s)|BP=Io1Vin=−s((gm1gm2)/C1)D(s)T2(s)|LP=Io2Vin=(gm1gm3gm4)/C1C2D(s)T3(s)|HP=Io3Vin=s2gm1D(s)T4(s)|NOTCH=(Io3+Io2)Vin=gm1(s2+gm3gm4/C1C2)D(s)T5(s)|AP=(Io3+Io2+Io1)Iinhhhhhhh=gm1{s2−s(gm2/C1)+gm3gm4/C1C2}D(s),

where

(3) $$\begin{array}{c} D\left(s\right)=s^{2}+s\left(\frac{g_{m_{1}}}{C_{1}}\right)+\frac{g_{m_{3}}g_{m_{4}}}{C_{1}C_{2}}. \end{array}$$

The *ω*_0_, BW, and quality factor (*Q*_0_) are given by

(4) $$\begin{array}{c} \omega_{0}=\sqrt{\frac{g_{m_{3}}g_{m_{4}}}{C_{1}C_{2}}} \end{array}$$

(5) $$\begin{array}{c} \text{BW}=\frac{g_{m_{1}}}{C_{1}} \end{array}$$

(6) $$\begin{array}{c} Q_{0}=\sqrt{\frac{g_{m_{3}}g_{m_{4}}C_{1}}{{\left(g_{m_{1}}\right)}^{2}C_{2}}}. \end{array}$$

From (4) and (5), it is seen that *ω*_0_ and BW are independently controllable, the former through *g*_*m*_3__ or *g*_*m*_4__ or *C*_2_ and the later through *g*_*m*_1__.

*I*_*o*1_ and *I*_*o*2_ are explicit current outputs but *I*_*o*3_ is taken through grounded capacitor *C*_1_. To extract this current (*I*_*o*3_) explicitly another device with its input virtually grounded will be needed due to which the capacitor *C*_1_ will not be physically connected to ground but it will still be virtually grounded [11].

## 3. Nonideal Analysis and Sensitivity Performance

Taking into account the various VDTA parasitics such as the finite* P*-terminal parasitic impedance consisting of a resistance *R*_*P*_ in parallel with capacitance *C*_*P*_, the finite* N*-terminal parasitic impedance consisting of a resistance *R*_*N*_ in parallel with capacitance *C*_*N*_, the finite* X*-terminal parasitic impedance consisting of a resistance *R*_*X*_ in parallel with capacitance *C*_*X*_, and the parasitic impedance at the* Z*-terminal consisting of a resistance *R*_*Z*_ in parallel with capacitance *C*_*Z*_, then the *ω*_0_ and *Q*_0_ including the influence of parasitic are given by

(7) ω0=((2RzRn+1RxRz+1Rz2+gm1Rz+gm3gm4)   ×(Cz2C1C2+2CnC2+CxC2+CzC2+C1Cz      +CxCz+2CnCz+Cz2)−12RzRn)1/2Q0=(2RzRn+1RxRz+1Rz2+gm1Rz+gm3gm4)   ×(Cz2C1C2+2CnC2+CxC2+CzC2+C1Cz      +CxCz+2CnCz+Cz2)1/2   ×(2C2Rn+C2Rx+C2Rz+C2gm1+2CzRn+CzRx      +2CzRz+Czgm1+C1Rz+2CnRz+CxRz)−1.

Using the classical definition of the sensitivity coefficient, that is,

(8) $$\begin{array}{c} S_{x}^{F}=\frac{x}{F}\frac{\partial F}{\partial x}, \end{array}$$

where “*F*” may represent the parameters of the elements and “*x*” may represent passive elements as well as the active elements with respect to which the sensitivity is to be evaluated and by taking *C*_1_ = 0.05 nF, *C*_2_ = 0.1 nF, *R*_*p*_ = *R*_*z*_ = *∞*, *C*_*p*_ = *C*_*x*_ = *C*_*z*_ = 0, *g*_*m*_1__ = *g*_*m*_2__ = *g*_*m*_3__ = *g*_*m*_4__ = 631.702 *μ*A/V, the sensitivities of *ω*_0_ and *Q*_0_ are found to be either 0 or 0.5. Thus, all the active and passive sensitivities of *ω*_0_ and *Q*_0_ are low.

## 4. Simulation Results

**Figure 3:**
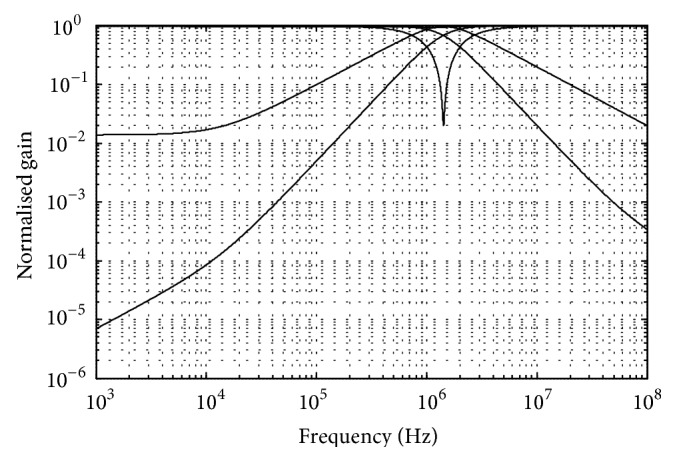
Frequency response of proposed TA filter.

To confirm theoretical analysis, the proposed configuration was simulated using CMOS VDTA as given in [8]. The passive elements of the configuration were selected as *C*_1_ = 0.05 nF and *C*_2_ = 0.1 nF. The power supplies used for CMOS VDTA were ±0.9 V. The transconductances of VDTA were controlled by bias currents.  Figure 3 shows the simulated filter responses of LP, BP, HP, BR, and AP. A comparison with previously published TA biquad is shown in Table 1. These results, thus, confirm the validity of the proposed configuration.

## 5. Conclusion

A new Transadmittance-mode biquad filter has been proposed which employs only two VDTAs and two grounded capacitors. The proposed filter can realize the second-order LP, BP, and HP responses simultaneously without changing the circuit topology and without any matching condition. The notch and AP filter responses are also obtainable through proper connections of the currents. The filter circuit offers (i) independent control of *ω*_0_ and BW and (ii) low active and passive sensitivities. SPICE simulations have established the workability of the proposed formulation.

## Figures and Tables

**Table 1:** 

Reference	Active elements used	Number of resistors	Number of capacitors	Number of simultaneously realized filters
[3]	1	2 (1G + 1F)	1G	BP, LP
[2]	3	2 (1G + 1F)	2F	BP, LP, HP
[1]	3	3 (1G + 2F)	2F	BP, LP, HP
Proposed	2	0	2G	BP, LP, HP

^*^G: grounded, F: floating.
